# Correction: Graphene material preparation through thermal treatment of graphite oxide electrochemically synthesized in aqueous sulfuric acid

**DOI:** 10.1039/c8ra90043d

**Published:** 2018-05-24

**Authors:** B. Gurzęda, T. Buchwald, M. Nocuń, A. Bąkowicz, P. Krawczyk

**Affiliations:** Institute of Chemistry and Technical Electrochemistry, Poznan University of Technology Berdychowo 4 60-965 Poznań Poland piotr.krawczyk@put.poznan.pl; Faculty of Technical Physics, Poznan University of Technology Piotrowo 3 60-965 Poznań Poland; Faculty of Materials Science and Ceramics, AGH University of Science and Technology al. Mickiewicza 30 30-059 Kraków Poland

## Abstract

Correction for ‘Graphene material preparation through thermal treatment of graphite oxide electrochemically synthesized in aqueous sulfuric acid’ by B. Gurzęda *et al.*, *RSC Adv.*, 2017, **7**, 19904–19911.

The authors regret that the incorrect project number as financial support was included in the original article. The correct project number is 2015/17/B/ST8/00371.

The Royal Society of Chemistry apologises for these errors and any consequent inconvenience to authors and readers.

## Supplementary Material

